# On the Alleged Production of Phosphate of Lime and Iron in the Egg during Incubation

**Published:** 1849-06

**Authors:** Alfred S. Taylor


### CHEMISTRY.

*On the alleged production of Phosphate of Lime and Iron in the Egg during incubation.* By Alfred S. Taylor, F. R. S.—In a work published some years since, Sir Gilbert Blane, while treating of the assimilative principle, says, that one of its properties is to reproduce, or actually create substances which are commonly regarded by chemists as simple or elementary. Among the illustrations which he brings forward in support of his statement are the following:—1. That the flesh and bones, for instance, of an ox, an animal subsisting on pure vegetable food ; of a lion, an animal subsisting on pure animal food ; and of a hog, an animal subsisting on mixed food ; though differing in some of their sensible qualities, are identical when considered as chemical compounds. 2. He regards it as a curious and inexplicable question, how it happens that azote enters as much into the composition of the flesh of graminivorous and herbivorous animals, in whose food no azote is found, as it does into the flesh of carnivorous animals, in which this principle abounds. "There is none in the food of the former, and it appears, by experiment, that no azote (nitrogen) is absorbed from the respired air. Not only, therefore, is none abstracted from the air, but some must have been generated by the powers of life. 3. More carbon is daily eliminated in the process of human respiration in twenty-four hours than can have been supplied by the aliment, whether vegetable or animal. 4. Phosphorus is an essential constituent of bone: it is found in the urine, and abounds particularly in that of the horse, though there is little or none in his food. 5. The production of coral, madrepore, chalk, &c., consisting of the exuviæ of shell-fish. It is impossible that this mass of matter could have preëxisted in the food of testaceous and crustaceous animals. 6. The bones of the chick far advanced in incubation contain phosphate of lime, although neither phosphorus nor lime is discoverable in those parts of the egg from which it must have derived its nutriment. Iron is found in a newly-hatched chick, though *none can be detected in an egg before incubation.* 7. There are large quantities of phosphate of lime found in the shells of crustaceous animals.

Such are the physiological and chemical grounds upon which the ingenious writer referred to, bases his argument in support of the view, that what are denominated elementary substances are created in the animal body under the influence of the vital principle. It is not my intention to affirm that all the substances known to chemists as simple bodies are insusceptible of being reproduced under some unknown conditions. It is not improbable that, as science advances, facts establishing this may be brought to light; and it may hereafter become a subject of demonstration that some of these elements are compounds, and may thus be produced under the influence of the vital principle, or of electrical agency. In the meantime, however, it is right to examine carefully the postulates upon which an admission of this doctrine depends, and to see that we are not receiving mere figments of the mind in place of demonstrated truths. The greatest source of error in all inquiries concerning natural phenomena, is the tendency in the mind to generalize from a limited number of instances; and this tendency may be traced to the fact, that it is far less troublesome to reason upon general notions than to make experiments.

Since the above views were propounded by Sir Gilbert Blane, a most extraordinary advance has been made in organic chemistry; and what then appeared as incomprehensible difficulties, except upon the theory for the support of which they were adduced, are now, for the most part, easily susceptible of explanation. To the researches made by the German School of Chemists, the science is much indebted for the discovery of numerous important and interesting facts, which have established the existence of closely connecting links between the animal and vegetable kingdoms.

Thus the identity in the composition of muscle, whether taken from a carnivorous or herbivorous animal, offers no longer a difficulty to the chemical physiologist. Albumen, casein, and oil exist in the vegetable as well as in the animal tissue; and the fibrine of the animal kingdom has its analogue in the gluten of the vegetable. It is not here necessary to consider whether the proportions of these principles in the two kinds of food be the same, or whether the principles themselves contain precisely the same amount of nitrogen, sulphur, or phosphorus, either free or under the form of phosphates; nor is it necessary to admit the correctness of the hypothetical formulæ of Mulder and Liebig, in order to justify us in saying that this part of the argument for the reproductive power of the so-called assimilative principle has been entirely overthrown by the discoveries of modern science. It is, therefore, now no longer curious or inexplicable that nitrogen should be a large constituent of muscle, or phosphorus of bone and urine, in vegetable feeders.

Although physiologists still differ concerning the source and quantity of carbon eliminated in respiration, there is no reason whatever to suppose that is formed within the body, or that its quantity is greater than that which would result from the detritus of the organs or the carbon of the aliment taken by the animal. Nor does the production of coral or madrepore in the bed of the ocean, or of pearl in the shell of the oyster, bear out the opinion for which this is adduced as one example of reproduction. Sea-water contains sufficient lime to allow of this material being converted by the work of polypi into submarine coral banks of enormous extent; and if we cannot fully explain the mode in which the choloride of calcium is thus brought to the state of carbonate of lime in the animal system, it is unnecessary to resort to the hypothesis that the material lime itself is created. It is also possible that phosphorus may exist in certain crustaceous fish; but is the introduction of this element, through the food of such fish, so excluded as to render it necessary to resort to the hypothesis of its production? It appears to me that it is not: and I would add, that, on examining the shells of the crustacea, ancient and modern, I have not detected any traces of phosphate of lime.

*Chemical Analysis of Animal and Vegetable Principles.*

*Gluten and Fibrine.*—Independently of elementary composition, there can be no doubt that these principles bear a close resemblance to each other in all their chemical relations. I have prepared gluten from flour by the usual process of washing, until it was no longer rendered purple or blue by water of iodine, and then have found that—

1. When boiled in pure and concentrated muriatic acid it became dissolved, forming a dark purple solution, and when this solution was added to a large quantity of water, the greater part of the gluten was again separated as a light-coloured precipitate.

2. When boiled with a solution of potash, containing, dissolved, a small quantity of oxide of lead, it acquired a dark brown colour, indicating the presence of sulphur.

3. When dried and heated, ammonia was abundantly evolved, indicating the presence of nitrogen.

4. It is soluble in caustic potash or soda, and in strong acetic acid.

In all these properties, vegetable gluten is not to be distinguished from animal fibrine.

*Vegetable and Animal Albumen and Casein.*—It is well known that the albumen of the egg, when boiled with pure and concentrated muriatic acid, acquires a rich pink, red, or purple colour. I have found the same effect to follow with the substance of the cocoa-nut, the hard kernel of the date, the almond, and other seeds, and also with the transparent and opaque part of feathers, as well as with horn and the human nail. Animal albumen, whether as feather, nail, or horn, produces, with potash and oxide of lead, a large quantity of sulphuret. Precisely the same result is obtained with the cocoa-nut, almond, and other vegetable seeds. The casein of the vegetable kingdom, as it is obtained from pease, possesses all the chemical properties of that derived from milk. There is the same amount of sulphur and nitrogen.

These facts, then, as well as the results of elementary analysis, without resorting to the hypothesis of Mulder—that all these principles are but modifications of one original substance, protein—leave it undoubted that the differences between animal and vegetable food in their constituent principles are more physical than chemical: and that in the vegetable kingdom are found all those simple substances which a few years ago were supposed to be produced by the animal organism. To Liebig is due the merit of having called especial attention to these facts. I have so far verified his statements by experiments, in order to show that the hypothesis of the production of sulphur, phosphorus, nitrogen, and carbon in the body is unsound. The chemical facts admit of another and more reasonable explanation.

The statement which appears most strongly to favour the argument for the production of elementary substances under the influence of the vital principle, is that which relates to the alleged formation of phosphate of lime and iron in the egg during incubation. If this view were correct, it would leave the question settled in the affirmative; for it is impossible to conceive that iron should traverse the pores of the shell during incubation, in order to become fixed in the body of the chick. Such an hypothesis would present even greater difficulties than that of its supposed formation.

I therefore resolved to put this question to the test of experiment, and a favourable opportunity having occurred, the following results were obtained. The albumen of the yolk of the egg is well known to be identical with that of the white. The yolk contains a yellow oil, separable by alcohol or ether, and according to Liebig, cholesterine and iron. It is stated in some of our best works on chemistry that iron is contained in the yolk at all periods, and it is implied that it is absent from the white.

1. Two eggs were boiled for some minutes to coagulate the albumen, and the whites and yolks were then carefully separated and thoroughly dried. Each mass was then separately incinerated in a clean platina crucible, and the ash digested in pure muriatic acid (free from iron) carefully distilled for the purpose, and diluted with a small quantity of water. The liquid was filtered, and tested with the ferro-cyanide of potassium and sulpho-cyanide of potassium. A precipitate of prussian blue was obtained with the former, and the deep red colour, indicative of a persalt of iron, was the result of the addition of the latter. The same effects were produced in acting upon the ash in both cases; whence it was inferred that iron was not only present in the white of egg, but its quantity appeared to be as nearly as possible the same as that which existed in the yolk.

2. In this experiment a perfectly fresh-laid egg was taken. It was boiled for some minutes to perfect coagulation, and the shell was carefully separated from the adhering membranes.

*The Shell.*—This was dissolved in diluted muriatic acid: there was copious effervescence, and some animal matter was set free during the process of solution. The muriatic acid was so adjusted as not to be more than sufficient to dissolve the earthy basis. On adding ammonia there was a very faint milky precipitate; and on adding phosphate of ammonia and ammonia there was a slight gelatinous precipitate, which did not disappear entirely on supersaturating with muriate of ammonia; and lastly, on adding the ferro-cyanide and sulpho-cyanide of potassium there was no change. From these results I infer that the shell consisted of animal matter, of carbonate of lime in large proportion, faint traces of phosphate of lime, and probably of magnesia, but no portion of iron.

The yolk and white, including the membranes, were then dried and incinerated; the ash digested, as in the before-mentioned experiment, in pure muriatic acid diluted with water; and this solution after filtration, was tested—1st, by ammonia, which threw down a very copious gelatinous precipitate of phosphate of lime; 2dly, by the ferro-cyanide of potassium, which produced a perceptible quantity of prussian blue; and 3dly, by sulpho-cyanide of potassium, which produced the deep red colour of the sulpho-cyanide of iron. These results were compared with the action of the tests separately upon the muriatic acid employed, in order to ensure the purity of the solution used; and the differences were such as to lead clearly to the inference that *iron and phosphate of lime are large constituents of the recently-laid egg.*

3. For this experiment I procured an egg which had reached the twenty-second day of incubation. The shell had been broken in the usual situation, but the chicken, which was perfectly developed, was dead. The shell, with the chicken contained in it, was in the first instance dried by a gentle heat. The shell was entirely separated, and the only difference between this and the recent shell was, that it was much more brittle, and more easily reduced to a fine powder. It was dissolved in diluted muriatic acid, and the results obtained by applying tests to the solution were the same as those described under 2. It was impossible to perceive any chemical difference between the recent and the incubated shell.

The chicken being divided into fragments, and the yolk contained in its abdomen thoroughly dried, the whole mass was incinerated in a platina crucible, and the ash digested, as in 2, in diluted muriatic acid. The same tests were applied, with results so perfectly simllar as to render it impossible for an indifferent person to say which was the ash of the recent, and which of the incubated egg. The research was directed chiefly to the presence of iron and phosphate of lime; and both of these bodies were present, but not in greater quantity than had been found to exist in the recent egg. A direct quantitative analysis was not performed, but the circumstances in the two cases were made as nearly as possible the same, by taking equal quantities of solutions in the same acid liquid, and using like quantities of the tests. After all, the proportions of iron and phosphate of lime contained in the egg, whether recent or old, are small, and the principal object of the inquiry was rather to determine whether iron and phosphate of lime were or were not constituents of the recent egg.

From these experiments and observations I would therefore draw the following conclusions:—

1. That the chemical composition of vegetable food is such as readily to account for the introduction of carbon, phosphorus, sulphur, and nitrogen into the animal system, and to render it unnecessary to assume that these elementary principles are products of the animal organism.

2. That the fluid parts of the egg contain iron and phosphate of lime in some unknown state of combination at all periods.

3. That iron and phosphate of lime are contained in the freshly-laid egg, as well as in that which has undergone the process of incubation; and that from the application of tests in quantitative analysis, there is no reason to believe that the proportions of these two bodies are greater at one time than at another.

4. That iron is contained in the albumen as well as in the yolk of the egg, and probably in equal proportion.

5. That the shell of the egg contains no iron, and but very slight traces of phosphate of lime.

The illustrious Boerhaave, writing in 1727, says, in reference to this subject: "All the parts of a chick, as the blood, flesh, bones, &c., are formed out of the bare white of the egg (albumen); for nothing but this is consumed during the time of the incubation of the hen, the yolk all the while remaining entire." Liebig, writing in 1842, says: "Yet we see in the process of incubation, during which no food and no foreign matter, except the oxygen of the air, is introduced or can take part in the development of the animal, that out of the albumen,—feathers, claws, globules of the blood, fibrine, membrane and cellular tissue, arteries and veins, are produced." It will thus be seen that a century has added but little to our knowledge of this subject; for Boerhaave has arrived at the same conclusion to which a more correct analysis has led modern chemists. That the albumen or white is principally concerned in these singular changes is proved by the fact that the greater part of the yolk is found entire within the body of the chick towards the last stage of incubation. We have, then, during this process, under the influence of a vital principle, no creation of elementary matter in the egg; but a most marvellous instance of the re-arrangement of the principles which it contains in new physical forms; the conversion of soluble into insoluble albumen, under the form of feathers, claws, and cellular membrane, with the concentration of the phosphate of lime in bone, and of the iron in the blood.—*Guy's Hospital Reports.*

